# Psychometric Properties of Scales Measuring Resilience in U.S. Latinx Populations: A Systematic Review

**DOI:** 10.1089/heq.2022.0123

**Published:** 2023-03-03

**Authors:** Joshua D. Cockroft, Julia Rabin, R. Andrew Yockey, Isabella Toledo, Susan Fain, Farrah Jacquez, Lisa M. Vaughn, Shanna D. Stryker

**Affiliations:** ^1^ Department of Family and Community Medicine, University of Cincinnati College of Medicine, Cincinnati, Ohio, USA.; ^2^ Department of Psychiatry and Behavioral Neurosciences, University of Cincinnati College of Medicine, Cincinnati, Ohio, USA.; ^3^ Department of Psychology, University of Cincinnati College of Arts and Sciences, Cincinnati, Ohio, USA.; ^4^ Department of Biostatistics and Epidemiology, The University of North Texas Health Science Center at Fort Worth, Fort Worth, Texas, USA.; ^5^ Department of Pediatrics, Cincinnati Children's Hospital Medical Center and University of Cincinnati College of Medicine, Cincinnati, Ohio, USA.; ^6^ School of Education, University of Cincinnati College of Criminal Justice, Education, and Human Services, Cincinnati, Ohio, USA.

**Keywords:** resilience, Latinx, psychometric, validation

## Abstract

**Objectives:**

Instruments used to measure resilience have typically been developed in European or Anglosphere countries and emphasize personal factors of resilience. In addition to being a quickly growing ethnic minority group in the United States, Latinx individuals face unique stressors and protective factors that may contribute to resilience. This review sought to determine the extent to which instruments measuring resilience have been validated in U.S. Latinx populations and what domains of resilience those scales capture.

**Methods:**

A systematic literature review was conducted using Preferred Reporting Items for Systematic Reviews and Meta-Analyses (PRISMA) standards and included studies describing psychometric properties of resilience scales for Latinx individuals living in the United States. Articles were assessed for quality of psychometric validation; scales used in the final studies were assessed for representation of domains of the social ecological resilience model.

**Results:**

Nine studies were included in the final review examining eight separate resilience measures. The populations of these studies were heterogeneous geographically and demographically; more than half the studies only included Latinx populations as a subgroup. The breadth and quality of psychometric validation were variable across studies. The domains represented by the scales in the review most heavily assessed individual domains of resilience.

**Conclusion:**

The literature to date on psychometric validation of resilience measures in Latinx populations in the United States is limited and does not robustly capture aspects of resilience that may be particularly meaningful for Latinx populations, such as community or cultural factors. Instruments that are developed with and for Latinx populations are necessary to better understand and measure resilience in this population.

## Introduction

Resilience is an individual's ability to successfully adapt during a stressful or adverse life event without a sustained, significant impact on the psychological or physical functioning.^1–3^ In general population studies, high levels of resilience are positively associated with life satisfaction and quality of life and negatively associated with a range of psychological disorders, including major depressive disorder, anxiety disorders, and post-traumatic stress disorder.^4–6^ Prior research suggests resilience is a modifiable and multidimensional construct that develops through a dynamic interaction between individual assets (e.g., self-efficacy, self-esteem, optimism) and environmental or community factors (e.g., peer support, access to resources, connections to one's culture).^3,7–9^

Currently, there is no single definition or theoretical basis unanimously agreed upon for resilience,^9,10^ which creates difficulty when developing relevant measurement tools and evaluating the impact of interventions and policy changes.^9,11^ As described in a review by Windle et al, psychometric properties are strong for scales such as the Connor–Davidson Resilience Scale (CD-RISC), the Resilience Scale for Adults (RSA), and the Brief Resilience Scale (BRS), but these scales focus more on individual assets and account less for societal and community domains. In addition, of the 15 resilience-measuring scales described by Windle et al, all but 2 (including the CD-RISC, RSA, and BRS) were developed in Europe, the United Kingdom, the United States, Australia, or Canada; it is uncertain whether these measures have predictive validity among culturally and socioeconomically diverse communities, particularly those outside of a Western context.^3,11–15^

Resilience studies focusing on individual characteristics may carry an implication of “blaming those who do not cope and exonerating the macro system of society from its responsibility to deal with adverse social conditions.”^16^ Indeed, existing individualistic scales and models of resilience may lack the perspective, values, and language necessary to understand resilience in marginalized and diverse groups due to an under-recognition of cultural differences in the expression of resilience and the complex interplay between minoritized communities and overarching environmental and societal factors.^17,18^

As an alternative to exclusively individualistic models of resilience, the social ecological approach to resilience (SER) model embraces an acknowledgment of the structural, social, and environmental factors relevant to wellness, and may better describe the context, opportunities, or threats relevant to resilience for minoritized populations. Inspired by Bronfenbrenner's bioecological model of human development and Masten's framework for resilience,^19,20^ the SER approach illuminates the interaction between a person's strengths and challenges, and the contextual ecology that may include protective and/or threatening social and environmental conditions, opportunities, and meaning-generating systems that influence wellness.^9,17,21^ Prior multinational studies conducted by the Resilience Research Center have identified 32 aspects of resilience relevant across 14 different cultural contexts that are both universal and culturally specific in nature.^17,22^

Within the SER model, these aspects broadly can be categorized by four overarching and interacting domains to produce resilience: (a) personal; (b) relational; (c) structural; and (d) spiritual/cultural. The personal domain includes individual traits and dispositions, such as self-esteem, that bolster ability to overcome adversity. The relational domain refers to the range of social networks (e.g., mentors, friends, family, peers, teachers) that care for an individual. The structural domain includes other environmental- or systems-level variables (e.g., social services, neighborhood safety, financial resources) that affect well-being, and the spiritual/cultural domain refers to relevant morals, values, and cultural practices that may positively contribute to well-being.^23^ Although these four domains reflect the interaction of diverse and heterogeneous contexts and social determinants of health, more research is necessary on the full range of constructs and measures relevant to the SER approach.^13,17,23,24^

Latinx individuals (those with origin or descent within Latin American countries) represent close to half of the total population growth of the United States between 2010 and 2020 but are less likely to be included in psychological research.^25,26^ The unique stressors of daily life as a minority in the United States place Latinx individuals at increased risk for poor physical and mental health outcomes, and are particularly salient for Latinx immigrants.^27–30^ Experiences of discrimination and heightened anti-immigrant rhetoric, for example, have been associated with chronic stress as well as depressive and anxious symptoms among Latinx youth and adults.^31,32^

Fortunately, studies have found that various spiritual and cultural constructs relevant to the experience of Latinx individuals in the United States can help protect against poor mental health outcomes. For example, constructs such as *familismo* (a trait of Latinx families that values the needs and wants of family as more important than one's own), religiosity, and biculturalism have been linked to fewer mental health symptoms among Latinx youth.^33,34^ These constructs are examples of the spiritual/cultural domain of SER that have demonstrated importance in Latinx groups. Unfortunately, it is unknown whether scales reflecting these domains have been created or validated in Latinx populations.

The aim of this article is to systematically review the psychometric properties of scales measuring resilience as studied in Latinx individuals living in the United States, and to describe the domains of resilience captured by these scales within an SER model of resilience. While acknowledging there may be further room for refinement and improvement of its use as a theoretical construct, the SER model was chosen for this review recognizing that traditional psychometric validation methods may not fully capture the validity of resilience scales as applied to a minoritized population. Having robust scales to describe resilience in a population that faces high levels of psychosocial stress and inequities can increase our understanding of resilience in this population and could better identify opportunities for evaluating community or clinical interventions.

## Methods

This literature review was guided by the Preferred Reporting Items for Systematic Reviews and Meta-Analyses (PRISMA) standards.^35^ The search was conducted June 23, 2021, using the following health and social science databases: PubMed, MEDLINE, PsycINFO, CINAHL, Scopus, and Embase. After each step of this review, consensus meetings were conducted between all reviewers involved to resolve discrepancies or disagreements in the study selection and data extraction process.

### Eligibility criteria

Peer-reviewed articles were eligible for this review if they met the following criteria: (a) study describes psychometric properties of a scale used to measure resilience; (b) study participants included Latinx individuals living in the United States or U.S. territories; (c) the scale was studied in English, Spanish, or Portuguese; and (d) full article was available in English. A study was considered to include Latinx individuals if it included a sample or subsample of participants living in the United States or U.S. territories who self-identified as “Hispanic,” “Latinx,” “Latino,” “Latina,” or “Latine” or identified with an ethnicity from any Latin American country. Multicountry studies were considered eligible if they included U.S. Latinx participants as defined above. Studies from any date/year available in each database were considered eligible for review.

Unpublished studies, dissertations, conference abstracts, theses, and studies not published in peer-reviewed journals were excluded from the present review. Gray literature (i.e., technical reports, fact sheets) was not consulted for the present review.

### Search

The search terms used for the databases are displayed in Table 1; in PubMed, the same search terms were used and relevant medical subject heading terms were included.

**Table 1.** Search Terms Used in Literature Review

Variable	Search terms	MeSH terms (included in PubMed search)
1	Resilien^*a^	Resilience, psychological
2	Hispanic OR Latin^*^	Hispanic Americans
3	Scale OR measure OR questionnaire OR survey OR inventor^*^ OR index OR checklist OR profile OR evaluat^*^	Surveys and Questionnaires
4	Psychometric OR valid^*^ OR reliab^*^ OR consistenc^*^ OR “factor analysis” OR evaluat^*^	Psychometrics
1 AND 2 AND 3 AND 4		

^a^ Asterisks were used when databases would allow this to include alternate endings, for example, Latin^*^ would capture Latino, Latinos, Latina, Latinas, Latine, Latinx.

MeSH, medical subject heading.

### Study selection process

All articles identified in the database searches were uploaded into Covidence (Covidence systematic review software, Veritas Health Innovation, Melbourne, Australia; available at www.covidence.org) to facilitate the selection process. Duplicates were removed by the software, and author S.D.S. reviewed a random selection of the identified duplicates to ensure accuracy of the software; there were no inaccuracies identified. The nonduplicate articles were divided evenly among three pairs of reviewers (J.D.C., J.R., A.Y., I.T., S.F., and S.D.S.) who screened the articles' titles and abstracts for relevancy. The relevant articles were then divided evenly among the same six reviewers but in reassigned pairs who screened the full-length articles for eligibility.

### Data extraction

A standardized extraction form was used to compile the following information for each included study: article title, scale/measure studied, language of the scale, study location, study date, and demographics of study participants. Two pairs of reviewers (J.D.C., J.R., A.Y., and S.D.S.) assessed the quality of each study's methodological approach to psychometric validation and the quality of specific psychometric properties of each scale used. Similar to the approach used by Windle et al,^3^ quality criteria were adapted from Terwee et al^36^ and Prinsen et al^37^ (Table 2) to ensure uniformity in interpretation and assessment by reviewers. Psychometric properties assessed included content validity, structural validity, internal consistency, construct validity, and criterion validity.

**Table 2.** Quality Assessment of Psychometric Properties of Each Scale

Property	Definition	Quality criteria
Content validity	The extent to which the domain of interest is comprehensively sampled by items in the questionnaire (that the measure represents all facets of the construct under question)	+ A clear description is provided of the measurement aim, the target population, the concepts that are being measured, and the item selection AND target population and (investigators OR experts) were involved in item selection; ? A clear description of abovementioned aspects is lacking OR only target population involved OR doubtful design or method; − No target population involvement; 0 No information found on target population involvement.
Structural validity	The degree to which the scores of a measure are an adequate reflection of the dimensionality of the construct	CFA: + A clear description of *a priori* factor models being tested AND CFI/TLI/comparable measure >0.95 OR RMSEA <0.06 OR SRMR <0.08 ? Clear description or justification is lacking − Clear description or justification present but CFI/TLI/comparable measure <0.95 OR RMSEA >0.06 OR SRMR >0.08 0 No information EFA: + A clear description of methods used to determine the number of factors that is methodologically sound (e.g., K1 rule, scree plot) AND >1 method used AND factor rotation obtained AND proposed factor structure model appears compatible with theoretical construct ? Clear description is lacking OR doubtful design or method − Proposed factor structure model does not appear compatible with theoretical construct but adequate methods 0 No information
Internal consistency	The extent to which items in a (sub)scale are intercorrelated, thus measuring the same construct	+ At least low evidence for structural validity AND adequate design and method AND Cronbach's alpha calculated between 0.70 and 0.95 ? Doubtful design and method or structural validity − At least low evidence for structural validity AND Cronbach's alpha(s) <0.70 or >0.95 OR inadequate design and method 0 No information found on internal consistency or structural validity
Construct validity	The extent to which scores on a particular questionnaire relate to other measures in a manner that is consistent with theoretically derived hypotheses concerning the concepts that are being measured	+ Specific hypotheses were formulated AND at least 75% of the results are in accordance with these hypotheses; ? Doubtful design or method (e.g., no hypotheses); − Less than 75% of hypotheses were confirmed, despite adequate design and methods; 0 No information found on construct validity
Criterion validity	The extent to which scores on a particular questionnaire relate to a gold standard	+ Convincing arguments that gold standard is “gold” AND correlation with gold standard ≥0.70; ? No convincing arguments that gold standard is “gold” OR doubtful design or method; − Correlation with gold standard <0.70, despite adequate design and method; 0 No information found on criterion validity

+ means high quality; ? means questionable quality; – means low quality; 0 no information reported.

CFA, confirmatory factor analysis; CFI, comparative fit index; EFA, exploratory factor analysis; RMSEA, root mean square error of approximation; SRMR, standardized root mean squared residual; TLI, Tucker-Lewis index.

### Corresponding item content to domains of resilience

Using a four-domain SER model, two reviewers (J.D.C. and J.R.) separately analyzed the items of each scale and determined which of the four domains each item fits best within (i.e., personal, relational, structural, or spiritual/cultural). The two reviewers reconciled differences in assignment of items to domains to produce a unanimous decision of how each included scale weighted particular domains of resilience.

## Results

**FIG. 1.**
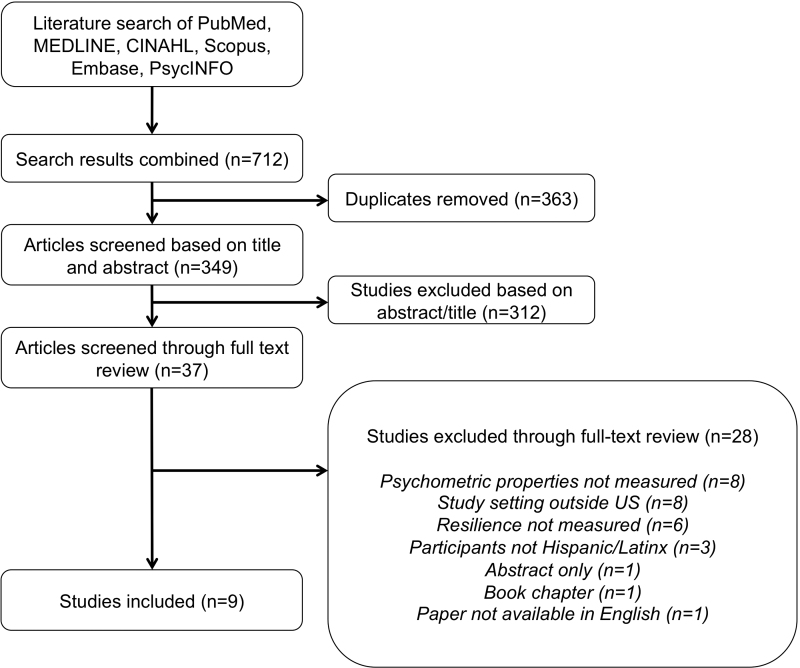
PRISMA flow diagram of study inclusion. PRISMA, Preferred Reporting Items for Systematic Reviews and Meta-Analyses.

A combined total of 712 abstracts published between 1997 and 2021 were found in a search of the 6 databases, as seen in Figure 1. Of these, 363 duplicate records were removed. The abstracts and titles of 349 articles were reviewed for relevance, of which 312 were excluded. The full text of 37 articles with relevant titles and abstracts were reviewed for full eligibility and an additional 28 studies were excluded, leaving 9 total studies included in the final review.

The nine included studies were published between 2003 and 2021, with data collected between 1998 and 2013 (although three studies did not clearly report the dates during which the data were collected). One study was conducted in Puerto Rico, and five were in geographic areas with historically large Latinx populations (defined here as areas outside of those that have historically large Latinx populations: Arizona, California, Colorado, Florida, Illinois, New Jersey, New Mexico, New York, or Texas).^38^ The other three were in “a Mountain West state,”^39^ an “urban county hospital,”^40^ and an “urban public school district in the northeastern U.S.”^41^ None was clearly in a nontraditional immigration destination (an area that has only within the last generation appreciated an increase in Latinx in-migration, and in which immigrants may face additional discrimination and barriers to services such as health care and education).^42^

Eight different scales were used across the nine studies (Table 3). This included the CD-RISC, Resilience Scale (RS), Health-Related Resilience Scale (HRRS), BRS, San Diego Risk and Resiliency Checkup (SDRRC), Benevolent Childhood Experiences (BCE) scale, Devereux Early Childhood Assessment Clinical Form (DECA-C), and the 2013 New Mexico High School Youth Risk and Resiliency Survey (NMHS-YRRS). The SDRRC was the only scale used in more than one study. The DECA-C and NMHS-YRRS include subscales measuring resilience within a larger instrument; psychometric properties of the relevant subscales only were analyzed for the purposes of this review.

**Table 3.** Reviewed Studies' Demographics and Psychometric Properties

Study, first author (year)	Scale	Language	*n*	Age range (years)	Population description	SES	Study date(s)	Content validity	Structural validity	IC	Construct validity	Criterion validity
Burrow-Sánchez (2014)^39^	Connor–Davidson Resilience Scale	Spanish English	106	13–18	Latinx adolescents with substance use disorders in a Mountain West state	Majority household annual income < $25,000	NR	−	+	+	+	0
Heilemann (2003)^44^	Resilience Scale	Spanish English	147	21–40	Latinx women of Mexican descent in urban Northern California	Majority monthly income <$1250	1998	−	−	+	+	0
Jimenez-Torres (2017)^47^	Health-Related Resiliency Scale	Spanish English	45	32–63	Puerto Rican HIV+ women in urban setting	Similar SES	2010–2011	+	0	+	+	0
Karaman (2019)^43^	Brief Resilience Scale	Spanish	193	18–58	Spanish-speaking undergraduate students in South Texas	NR	NR	−	+	+	+	0
Lee (2013)^46^	San Diego Risk and Resiliency Checkup	NR	Total: 2835 Latinx: 1838	12–18 (Latinx)	Youth offenders arrested by law enforcement under regular supervision in Los Angeles	NR	2008	0	+	+	?	0
Narayan (2018)^40^	Benevolent Childhood Experiences scale	Spanish English	Total: 101 Latinx: 37	18–44	Pregnant women planning to deliver at an urban county hospital	Patients of hospital serving low SES	NR	−	0	0	0	0
Oades-Sese (2010)^41^	Devereux Early Childhood Assessment Clinical Form	NR	410	3–5	Bilingual low-income Latinx American preschoolers in urban public school district in northeast United States	Eligible for free or reduced lunch	2008	+	−	+	0	0
Simmons (2021)^48^	2013 New Mexico High School Youth Risk and Resiliency Survey	NR	Total: 12,533 Latinx: 5827	14–17	Select New Mexico high school students	NR	2013	0	+	?	+	0
Turner (2006)^45^	San Diego Risk and Resiliency Checkup	English	Total: 1036 Latinx: 438	9–19+	Juvenile offenders served by four offices in the Los Angeles County Probation Department	NR	2002–2003	0	0	0	?	0

IC, internal consistency; NR, not reported; SES, socioeconomic status.

Most of the nine studies described properties of an existing scale in a Latinx population and/or studied properties of a Spanish translation.^39,41,43,44^ Two studies provided initial validation of an existing scale and included Latinx participants.^45,46^

Three studies described the development of new or adapted scales. The HRRS used an inductive approach to adapt the Resiliency Scales for Children and Adolescents to measure health-related resilience, and intentionally piloted it in women living with HIV in Puerto Rico.^47^ The BCE was developed based on prior work examining favorable childhood experiences that may promote resilience, as well as research on Adverse Childhood Experiences; it was developed with the intention for use in ethnically diverse and low-income samples.^40^ The NMHS-YRRS incorporated questions on resilience based on the California Healthy Kids Survey capturing “external resilience factors.”^48^

Between the 9 reviewed studies, a total of 17,406 participants from the United States and Puerto Rico were included, of whom 9041 identified as Latinx, Latine, Hispanic, Latina, or Latino. Ages of participants ranged from 3 to 63 years, with one study examining preschool students, four examining preadolescents or adolescents, and four examining adults. A wide breadth of populations were surveyed; examples include HIV+ women in Puerto Rico, pregnant women planning to deliver at an urban hospital, and Spanish-speaking undergraduate students in South Texas. Five of nine studies included participants from a lower income socioeconomic status bracket either exclusively or as a majority of participants. Five of nine studies directly targeted Latinx populations, while four targeted broader populations but included subgroup analysis for Latinx participants (Table 3). Among psychometric properties, criterion validity was the least reported, while internal consistency and structural validity were the most commonly described.

### Content validity

Of the nine studies, only two presented information on the content validity of and met the high-quality criteria for their scales by incorporating a clear description of the measurement aim, target population, concepts being measured, process of item selection, and participation of both experts and the target population in item selection. Jimenez-Torres et al utilized the Lawshe technique to calculate content validity indices (CVI) of their resiliency scale for HIV+ Latinx women (HRRS) through evaluation by 20 subject matter evaluators; CVI values for each item, subscale, and the overall scale (overall 0.62) were above the 0.42 cutoff for 20 evaluators.^47^ Oades-Sese et al described the involvement of experts and use of parent–teacher focus groups in the development of the DECA-C.^41^ Three studies described the influence of theory and expert input on the development of their respective scales, but did not describe involvement of the target population (CD-RISC, BRS, BCE).^39,40,43^

Heilemann et al present a clear description of their inclusion of members of the target population in the translation of the RS into Spanish, but do not clearly align the original theoretical concept to the lived experiences of the target population.^44^ The final three studies did not present information on assessing content validity of their scales (SDRCC, NMHS-YRRS).^45,46,48^

### Structural validity

Structural validity was more commonly well described than content validity. Four out of nine studies present factor analysis of their respective scales that demonstrates high quality.^39,43,46,48^ This includes a second-order single-factor structure for the BRS,^43^ a seven-item single-factor model of the CD-RISC derived by the authors,^39^ multigroup confirmatory factor analysis of the SDRRC demonstrating a first-order two-factor model with adequate fit through configural invariance and partial metric invariance indices across Caucasian, African American, and Latinx participants,^46^ and a single-factor model of the “resilience/interference” items for Latinx high school students on the NMHS-YRRS.^48^ Two studies, while methodologically sound, did not demonstrate good fit for their proposed hypothesized factor structures for their respective scales in their target populations (the RS and the DECA-C subscale “Total Protective Factors”).^41,44^ Three studies did not use factor analysis to determine the structural validity of their respective scales (BCE, HRRS and Turner and Fain's evaluation of the SDRRC).^40,45,47^

Exploratory factor analysis (EFA) or principal component factor analysis (PCFA) was conducted for two studies. However, Simmons et al did not extrapolate their EFA specifically to their Latinx high school student subgroup (SDRRC) while Heilemann et al's PCFA combined with varimax rotation did not allow for a clear interpretation of results (RS).^44,48^ Of note, while EFA or PCFA are most commonly used for new or adapted scales to determine the dimensionality of an instrument, this type of analysis was not used in the two studies that had adapted/developed the HRRS and BCE.^40,47^

### Internal consistency

Six out of nine studies reported a measure of internal consistency, Cronbach's alpha, that ranged from good to excellent for use in a Latinx group or subgroup (0.78 ⪙ α ⪙ 0.95) for their scales (CD-RISC, RS, HRRS, BRS, DECA-C, and Lee et al's evaluation of the SDRRC).^39,40,43,44,46,47^ Simmons et al reported an alpha value for its “Resilience-Interference” items (α=0.83) but this appears to have only been conducted for its overall population and not extrapolated specifically for the Latinx high-schooler subgroup (NMHS-YRRS).^48^ Two studies did not report internal consistency using Cronbach's alpha.

Turner and Fain present correlations between subscales of the SDRRC, but did not present interitem correlation or internal consistency; this analysis was also not extrapolated in subgroup analysis for Latinx participants.^45^ As an alternative measure of reliability, Narayan et al do present test–retest reliability of the BCE scale in pregnant women; this analysis demonstrated acceptable reliability for a subset of Latinx women (*r*=0.73, *p*<0.26, *n*=26).^40^

### Construct validity

Five studies clearly presented analysis of construct validity by testing hypotheses of resilience's correlation with other constructs for Latinx groups or subgroups. Three studies used instruments for depression (Beck Depression Inventory and Center for Epidemiologic Studies Depression scale) to measure theoretical convergent validity. Two of these studies demonstrated statistically significant negative correlation between depression and resilience, with small to medium strength of association (RS, HRRS).^44,47^ Burrow-Sánchez et al conducted a path analysis of a seven-item unidimensional factor of the CD-RISC to test a predictive relationship between this factor and depressive symptoms as mediated by ethnic identity (measured by the Multigroup Ethnic Identity Measure); this indirect effect was reported as negatively correlated and statistically significant.^39^ Heilemann et al also present a correlation between resilience and a single-item measure of “life satisfaction” that appeared to be created specifically for the authors' study (RS).^44^

Karaman et al describe a statistically significant and positive correlation between the BRS and the Short Grit Scale.^43^ Simmons et al proposed a hypothesized link between resilience and health behaviors (e.g., tobacco use, alcohol use); statistically significant correlations were noted for >75% of tested measures for their Latinx high schooler subgroup (NMHS-YRRS).^48^

Two studies presented analyses that were questionable as measurements of construct validity. Lee and colleagues present an analysis of convergent validity of the SDRRC. Pearson correlation coefficients were calculated only across subscales within the SDRRC, to the overall scale, and across subgroups, but not their Latinx subgroup.^46^ Turner and Fain also tested a hypothesized correlation between scores on the SDRRC and rates of recidivism for youth in the criminal justice system; the authors state that subgroup analysis based on race/ethnicity was done in correlation of recidivism and SDRRC scores and Latinx youth appeared to have relatively lower correlations in this comparison. However, the specific correlations are not clearly presented in either the narrative or published tables.^45^

Two studies did not present specific measures of construct validity including Latinx groups or subgroups. Narayan et al provide an analysis of theoretical convergent validity between BCE scores and adverse childhood experiences, post-traumatic stress disorder symptoms, and depressive symptoms, however, these correlations are not specifically presented for their Latinx subgroup.^40^ Oades-Sese et al acknowledge the need for further research to determine construct validity for the DECA-C.^41^

### Criterion validity

Given that researchers have not agreed on a uniform definition of resilience, and therefore there is no gold standard instrument against which to measure new instruments, no study was able to demonstrate criterion validity. As mentioned above, Karaman et al correlated the BRS to the Short Grit Scale and describe this as criterion validity,^43^ but because grit is distinct from resilience (grit specifically describes goal-directed perseverance), the criteria for criterion validity were not met.^49^

### Scales' relationship with construct of social ecological resilience

The authors of this review examined the eight scales from the nine included studies and matched each item to SER domains. All scales included items that reflected a “personal” domain of resilience; this was the most heavily emphasized domain in seven out of eight scales (the exception being the NMHS-YRRS). Three scales had items that exclusively measured “personal” aspects of resilience (BRS, DECA-C, and RS). Likewise, the number of items matching “personal” aspects of resilience constituted 71% of items across all scales. The “relational” domain was the second-most represented domain, with relevant items included in five out of eight scales; the number of items matching “relational” aspects of resilience included 20% of items across all scales. “Structural” and “spiritual/cultural” domains were less represented, present in only three and five scales, and with 7% and 3% of total item share, respectively (Table 4).

**Table 4.** Social Ecological Resilience as Measured in Scales

Scale used	Study referenced, first author (year)	Personal	Relational	Structural	Spiritual/cultural
San Diego Risk and Resiliency Checkup 60 items	Lee (2013)^46^; Turner (2006)^45^	+ (30)^a^	+ (19)	+ (9)	+ 2
Brief Resilience Scale 6 items	Karaman (2019)^43^	+ (6)	−	−	−
Devereux Early Childhood Assessment Clinical Form 62 items	Oades-Sese (2010)^41^	+ (62)	−	−	−
New Mexico High School Youth Risk and Resiliency Survey 15 items	Simmons (2021)^48^	+ (2)	+ (9)	+ (3.5)^b^	+ (0.5)^b^
Benevolent Childhood Experiences scale 10 items	Narayan (2018)^40^	+ (1)	+ (5)	+ (3)	+ (1)
Health-Related Resiliency Scale 30 items	Jimenez-Torres (2017)^47^	+ (18)	+ (11)	−	+ (1)
Resilience Scale 25 items	Heilemann (2003)^44^	+ (25)	−	−	−
Connor–Davidson Resilience Scale 25 items	Burrow-Sánchez (2014)^39^	+ (21)	+ (2)	−	+ (2)

^a^ The total number of items that were felt to represent a domain is in parentheses.

^b^ In this instance, a specific item was felt to represent both structural and cultural domains.

## Discussion

To our knowledge, this is the first study to review the psychometric properties and SER domains of scales measuring resilience in Latinx populations in the United States. This review identified only nine studies that validated resilience scales in this growing population, and almost half of the studies did so only through subgroup analysis. Eight scales were included among these studies, with only the SDRRC used in more than one study. A 2011 review showed that among general population samples, the three resilience scales with best-supported psychometric properties were the CD-RISC, the BRS, and the RSA.^3^ Of these, only the CD-RISC and BRS have been validated in U.S. Latinx populations.

While the RSA has been useful in assessing clinical change and validated in international settings, including Peru, it has not been studied in Latinx populations in the United States, which may represent a more culturally diverse group than that found in a single Latin American country.^50^ An important scale that has not been validated in Latinx populations in the United States is the Child and Youth Resilience Measure (CYRM). The CYRM was developed by an international team, which included team members from Colombia,^22^ and the scale has been validated in Spanish.^51^ The CYRM has been studied in young children from disadvantaged backgrounds in the United States,^52^ but subgroup analyses exploring the psychometric properties in Latinx youth have not specifically been measured and so could not be included in this review.

The quality of psychometric evaluation in studies found for this review was highly variable and limited by heterogeneity in populations and methodology. Of the scales assessed and reviewed, the CD-RISC, RS, and BRS had the highest quality psychometric properties. Although some psychometric properties were frequently assessed (internal consistency, factor structure), there were notable gaps in statistical validation including lack of assessing content validity and misuse of factor analysis for *de novo* instruments. The aims of most included studies align with the recognition of the importance of examining how previously generated instruments may be incomplete in regard to capturing specific populations' lived experiences, but there is still much wanting from the literature in understanding how to best define and measure resilience in Latinx populations, and how this may affect the validation of resilience scales.

Ungar defines resilience as “the capacity of individuals to navigate their way to health-sustaining resources, including opportunities to experience feelings of wellbeing, and a condition of the individual's family, community and condition to provide these health resources and experiences in culturally meaningful ways.”^17^ This definition acknowledges the four domains of SER. Unfortunately, there is a strong emphasis on the personal domain of resilience among the few scales that have included Latinx participants in validation studies that may not accurately reflect domains of resilience salient to this population. Unsurprisingly, of the studies included in our review, the scales acknowledging relational, community, and spiritual/cultural domains were those developed with an intention for use with multicultural groups, including the NMHS-YRRS that was adapted for use in a multiethnic high school population in New Mexico and the BCE that was developed for use in ethnically diverse, low-income pregnant women.^40,48^

Although qualitative literature on the emphasis of SER domains in Latinx individuals/communities is limited, studies to date suggest an emphasis on environmental factors over personal. For example, Buckingham and Brodsky identify relational (e.g., supporting one another within the Latinx community) and cultural (e.g., celebrating holidays, sharing food and cultural practices, sharing ethnic identity over experiences as an immigrant) components of resilience that help Latinx participants overcome adversity in the United States.^53^ Similarly, cultural identity and social connectedness were consistently associated with resilience in Latinx adolescents in Southern California.^54^ Latinx parents and their children also stress the necessity of the relational and the community domains as facilitators of success and resilience among Latinx youth, with specific suggestions of parental monitoring, involvement of other invested adults and role models, and community support.^55,56^

Quantitative studies of Mexican-origin adolescents and Latinx women also identified the salience of cultural values, such as familism and fatalism, in their experience of resilience.^57,58^ Having access to meaningful resources such as support groups, legal support, and health promoter training is an important source of community resilience for Latinx immigrant communities in Washington D.C.^59^ However, more in-depth qualitative exploration of this topic is necessary to understand how these different layers of resilience differ by gender and age. Furthermore, future research would benefit from the integration of this qualitative knowledge into the development and testing of culturally informed resilience measures.

## Limitations

This review focused on scales validated in Latinx individuals living within the United States, which is a heterogeneous group that includes immigrants who may face opportunities and challenges relevant to resilience that are distinct from those living in Latin America. However, scales studied in a particular Latin American country could be useful in subgroups of U.S. Latinx populations from that country or region, and so excluding those studies is a limitation of this review. In addition, the populations across the included studies were fairly heterogeneous across age range, geographic location, and specific characteristics, which makes making generalizable inferences difficult, particularly across an already heterogeneous group such as Latinx individuals in the United States. Most were done in geographic areas with high density of Latinx individuals.

Despite this, several included studies did not include large numbers of Latinx individuals. Of note, with this review's search criteria and databases used, potentially eligible studies only dated back to 1997, while included studies only dated back to 2003. The authors of this review suspect that this may be due to certain factors such as the U.S. Census not counting Latinx people as “individuals of Spanish/Hispanic origin” until the 1980s and the rapid growth of the Latinx population between 1980s and 1990s.^60,61^ A growth of research interest in and the more recent conceptualization of what constitutes a “Hispanic” or “Latino” population thus did not emerge until this time.

Likewise, the earliest validated psychometric instrument measuring resilience was first published in 1989, meaning use of resilience scales is limited to the past three decades.^3^ This being said, we cannot fully rule out the existence of other studies or research that may have been conducted earlier than the studies included in this review. In addition, different types of construct validity (e.g., predictive validity, retrospective validity) were not measured; future research is warranted on whether these scales measured all different types of validity, given their importance in construct validation.

## Conclusion

Overall, this review shows that the quality of psychometric validation of pre-existing scales for use in U.S. Latinx populations is mixed. Conceptually, while Ungar has demonstrated dimensions of resilience that can be useful across cultures, available scales do not reflect SER domains that may be salient in Latinx populations (namely, community and spiritual/cultural domains). Future research should aim to build on the work done by qualitative researchers to design scales that better reflect all four SER domains with a Latinx lens. This next step is both critical and timely, particularly given the increased rates of stress and discrimination faced by Latinx populations living within the United States, and the potential impact on health of these experiences.^62–65^

Should research or interventions targeting resilience be implemented in response to addressing these issues, this review highlights the limited foundation for use of psychometric resilience scales in this population and the need to adapt culturally relevant conceptualizations of resilience.

## Abbreviations Used

BCE: Benevolent Childhood Experiences
BRS: Brief Resilience Scale
CD-RISC: Connor–Davidson Resilience Scale
CFA: confirmatory factor analysis
CFI: comparative fit index
CVI: content validity indices
CYRM: Child and Youth Resilience Measure
DECA-C: Devereux Early Childhood Assessment Clinical Form
EFA: exploratory factor analysis
HRRS: Health-Related Resilience Scale
IC: internal consistency
MeSH: medical subject heading
NMHS-YRRS: New Mexico High School Youth Risk and Resiliency Survey
NR: not reported
PCFA: principal component factor analysis
PRISMA: Preferred Reporting Items for Systematic Reviews and Meta-Analyses
RMSEA: root mean square error of approximation
RS: Resilience Scale
RSA: Resilience Scale for Adults
SDRRC: San Diego Risk and Resiliency Checkup
SER: social ecological approach to resilience
SES: socioeconomic status
SRMR: standardized root mean squared residual
TLI: Tucker-Lewis index

## References

[1] Cano MÁ, Castro FG, De La Rosa M, et al. Depressive symptoms and resilience among hispanic emerging adults: Examining the moderating effects of mindfulness, distress tolerance, emotion regulation, family cohesion, and social support. Behav Med 2020;46(3–4):245–257; doi: 10.1080/08964289.2020.1712646 31935162 PMC7358125

[2] Osório C, Probert T, Jones E, et al. Adapting to stress: Understanding the neurobiology of resilience. Behav Med 2017;43(4):307–322; doi: 10.1080/08964289.2016.1170661 27100966

[3] Windle G, Bennett KM, Noyes J. A methodological review of Resilience Measurement Scales. Health Qual Life Outcomes 2011;9(1):8; doi: 10.1186/1477-7525-9-8 21294858 PMC3042897

[4] Hu T, Zhang D, Wang J. A meta-analysis of the trait resilience and mental health. Pers Individ Differ 2015;76:18–27; doi: 10.1016/j.paid.2014.11.039

[5] Bogaerts S, van Woerkom M, Erbaş Y, et al. Associations between resilience, psychological well-being, work-related stress and Covid-19 fear in forensic healthcare workers using a network analysis. Front Psychiatry 2021;12:936; doi: 10.3389/fpsyt.2021.678895 PMC8226029 34177662

[6] Stoffel JM, Cain J. Review of grit and resilience literature within health professions education. Am J Pharm Educ 2018;82(2):6150; doi: 10.5688/ajpe6150 29606705 PMC5869747

[7] Kunzler AM, Helmreich I, Chmitorz A, et al. Psychological interventions to Foster resilience in healthcare professionals. Cochrane Database Syst Rev 2020;2020(7):CD012527; doi: 10.1002/14651858.CD012527.pub2 PMC8121081 32627860

[8] Olsson CA, Bond L, Burns JM, et al. Adolescent resilience: A concept analysis. J Adolesc 2003;26(1):1–11; doi: 10.1016/S0140-1971(02)00118-5 12550818

[9] Ungar M. Social Ecologies and Their Contribution to Resilience. In: The Social Ecology of Resilience: A Handbook of Theory and Practice. (Ungar M. ed.) Springer: New York, NY; 2012; pp. 13–31.

[10] Leppin AL, Gionfriddo MR, Sood A, et al. The efficacy of resilience training programs: A systematic review protocol. Syst Rev 2014;3(1):20; doi: 10.1186/2046-4053-3-20 24602236 PMC3946765

[11] Jongen C, Langham E, Bainbridge R, et al. Instruments for measuring the resilience of indigenous adolescents: An exploratory review. Front Public Health 2019;7:194; doi: 10.3389/fpubh.2019.00194 31380334 PMC6647871

[12] Ungar M. Nurturing hidden resilience in at-risk youth in different cultures. J Can Acad Child Adolesc Psychiatry 2006;15(2):53–58. 18392194 PMC2277285

[13] Ungar M. Researching and theorizing resilience across cultures and contexts. Prev Med 2012;55(5):387–389; doi: 10.1016/j.ypmed.2012.07.021 22884666

[14] Kirmayer LJ, Sehdev M, Whitley R, et al. Community resilience: Models, metaphors and measures. Int J Indig Health 2009;5(1):62–117; doi: 10.3138/ijih.v5i1.28978

[15] Blackwell M, Boj Lopez F, Urrieta L. Special issue: Critical Latinx indigeneities. Lat Stud 2017;15(2):126–137; doi: 10.1057/s41276-017-0064-0

[16] van Breda AD, Theron LC. A critical review of South African child and youth resilience studies, 2009–2017. Child Youth Serv Rev 2018;91:237–247; doi: 10.1016/j.childyouth.2018.06.022

[17] Ungar M. Resilience across cultures. Br J Soc Work 2006;38(2):218–235; doi: 10.1093/bjsw/bcl343

[18] Ungar M. Introduction to the Volume. In: The Social Ecology of Resilience: A Handbook of Theory and Practice. (Ungar M. ed.) Springer: New York, NY; 2012; pp. 1–9.

[19] Bronfenbrenner U, Evans GW. Developmental science in the 21st century: emerging questions, theoretical models, research designs and empirical findings. Soc Dev 2000;9(1):115–125; doi: 10.1111/1467-9507.00114

[20] Masten AS, Powell JL. A Resilience Framework for Research, Policy, and Practice. In: Resilience and Vulnerability: Adaptation in the Context of Childhood Adversities. (Luthar SS, ed.) Cambridge University Press; 2003; pp. 1–21.

[21] Ungar M. The social ecology of resilience: Addressing contextual and cultural ambiguity of a nascent construct. Am J Orthopsychiatry 2011;81(1):1–17; doi: 10.1111/j.1939-0025.2010.01067.x 21219271

[22] Ungar M, Liebenberg L. Assessing resilience across cultures using mixed methods: Construction of the child and youth resilience measure. J Mixed Methods Res 2011;5(2):126–149; doi: 10.1177/1558689811400607

[23] Vaughn LM, DeJonckheere M. The opportunity of social ecological resilience in the promotion of youth health and wellbeing: A narrative review. Yale J Biol Med 2021;94(1):129–141. 33795989 PMC7995941

[24] Hamby S, Taylor E, Smith A, et al. New measures to assess the social ecology of youth: A mixed-methods study. J Community Psychol 2019;47(7):1666–1681; doi: 10.1002/jcop.22220 31332818

[25] Bureau UC. 2020 Census Illuminates Racial and Ethnic Composition of the Country; 2021. Available from: https://www.census.gov/library/stories/2021/08/improved-race-ethnicity-measures-reveal-united-states-population-much-more-multiracial.html [Last accessed: May 20, 2022].

[26] Jacquez F, Vaughn L, Zhen-Duan J, et al. Health care use and barriers to care among Latino immigrants in a new migration area. J Health Care Poor Underserved 2016;27(4):1761–1778; doi: 10.1353/hpu.2016.0161 27818437

[27] Rodriguez DX, Hill J, McDaniel PN. A scoping review of literature about mental health and well-being among immigrant communities in the United States. Health Promot Pract 2021;22(2):181–192; doi: 10.1177/1524839920942511 32729336

[28] Ryan D, Tornberg-Belanger SN, Perez G, et al. Stress, social support and their relationship to depression and anxiety among Latina immigrant women. J Psychosom Res 2021;149:110588; doi: 10.1016/j.jpsychores.2021.110588 34371256 PMC8453089

[29] Cariello AN, Perrin PB, Morlett-Paredes A. Influence of resilience on the relations among acculturative stress, somatization, and anxiety in Latinx immigrants. Brain Behav 2020;10(12):e01863; doi: 10.1002/brb3.1863 32990393 PMC7749538

[30] Patler C, Hamilton ER, Savinar RL. The limits of gaining rights while remaining marginalized: The deferred action for childhood arrivals (DACA) program and the psychological wellbeing of Latina/o undocumented youth. Soc Forces 2021;100(1):246–272; doi: 10.1093/sf/soaa099

[31] Valentín-Cortés M, Benavides Q, Bryce R, et al. Application of the minority stress theory: Understanding the mental health of undocumented Latinx immigrants. Am J Community Psychol 2020;66(3–4):325–336; doi: 10.1002/ajcp.12455 32776579

[32] Ramos G, Ponting C, Bocanegra E, et al. Discrimination and internalizing symptoms in rural Latinx adolescents: The protective role of family resilience. J Clin Child Adolesc Psychol 2022;51(6):997–1010; doi: 10.1080/15374416.2021.1923018 34038290

[33] Marsiglia FF, Kulis S, FitzHarris B, et al. Acculturation gaps and problem behaviors among U.S. Southwestern Mexican Youth. Soc Work Forum (N Y N Y) 2009;42–43:6–26. PMC3719159 23888125

[34] DeJonckheere MJ, Vaughn LM, Jacquez F. Latino immigrant youth living in a nontraditional migration city: a social-ecological examination of the complexities of stress and resilience. Urban Educ 2017;52(3):399–426; doi: 10.1177/0042085914549360

[35] Moher D, Liberati A, Tetzlaff J, et al. Preferred reporting items for systematic reviews and meta-analyses: The PRISMA statement. BMJ 2009;339(5):b2535-b2535; doi: 10.1136/bmj.b2535 19622551 PMC2714657

[36] Terwee CB, Bot SDM, de Boer MR, et al. Quality criteria were proposed for measurement properties of health status questionnaires. J Clin Epidemiol 2007;60(1):34–42; doi: 10.1016/j.jclinepi.2006.03.012 17161752

[37] Prinsen CAC, Mokkink LB, Bouter LM, et al. COSMIN guideline for systematic reviews of patient-reported outcome measures. Qual Life Res 2018;27(5):1147–1157; doi: 10.1007/s11136-018-1798-3 29435801 PMC5891568

[38] Passel JS, Lopez MH, Cohn D. U.S. Hispanic Population Continued Its Geographic Spread in the 2010s; February 22, 2022. Available from: https://www.pewresearch.org/fact-tank/2022/02/03/u-s-hispanic-population-continued-its-geographic-spread-in-the-2010s/ [Last accessed: September 16, 2022].

[39] Burrow-Sánchez JJ, Corrales C, Jensen CO, et al. Resilience in a sample of Mexican American adolescents with substance use disorders. Psychol Assess 2014;26(3):1038–1043; doi: 10.1037/pas0000011 24932645 PMC4152411

[40] Narayan AJ, Rivera LM, Bernstein RE, et al. Positive childhood experiences predict less psychopathology and stress in pregnant women with childhood adversity: A pilot study of the benevolent childhood experiences (BCEs) scale. Child Abuse Negl 2018;78:19–30; doi: 10.1016/j.chiabu.2017.09.022 28992958

[41] Oades-Sese GV, Kaliski PK, Weiss K. Factor structure of the devereux early childhood assessment clinical form in low-income Hispanic American bilingual preschool children. J Psychoeduc Assess 2010;28(4):357–372; doi: 10.1177/0734282910366842

[42] Waters MC, Jiménez TR. Assessing immigrant assimilation: New empirical and theoretical challenges. Annu Rev Sociol 2005; 31(1):105–125; doi: 10.1146/annurev.soc.29.010202.100026

[43] Karaman MA, Cavazos Vela J, Aguilar AA, et al. Psychometric properties of U.S.-Spanish versions of the grit and resilience scales with a Latinx population. Int J Adv Counselling 2019;41(1):125–136; doi: 10.1007/s10447-018-9350-2

[44] Heilemann MV, Lee K, Kury FS. Psychometric properties of the Spanish version of the Resilience Scale. J Nurs Meas 2003;11(1):61–72; doi: 10.1891/jnum.11.1.61.52067 15132012

[45] Turner S, Fain T. Validation of the risk and resiliency assessment tool for juveniles in the Los Angeles county probation system. Fed Probat 2006;70(2):49–57.

[46] Lee S-Y. Testing psychometric properties and the cross-ethnic construct validity of the risk and resiliency checkup. Youth Violence Juv Justice 2013;11(2):165–177; doi: 10.1177/1541204012460875

[47] Jimenez-Torres GJ, Wojna V, Rosario E, et al. Assessing health-related resiliency in HIV+ Latin women: Preliminary psychometric findings. PLoS One 2017;12(7):e0181253; doi: 10.1371/journal.pone.0181253 28723939 PMC5517021

[48] Simmons JD, Smith JE, Erickson SJ, et al. A factor analytic approach to understanding health risk behaviors and resilience among multi-racial/ethnic adolescents in New Mexico. Ethn Health 2022;27(7):1652–1670; doi: 10.1080/13557858.2021.1925227 33971771

[49] Duckworth AL, Peterson C, Matthews MD, et al. Grit: Perseverance and passion for long-term goals. J Pers Soc Psychol 2007;92(6):1087–1101; doi: 10.1037/0022-3514.92.6.1087 17547490

[50] Morote R, Hjemdal O, Martinez Uribe P, et al. Psychometric properties of the Resilience Scale for Adults (RSA) and its relationship with life-stress, anxiety and depression in a Hispanic Latin-American community sample. PLoS One 2017;12(11):e0187954; doi: 10.1371/journal.pone.0187954 29125876 PMC5681258

[51] Llistosella M, Gutiérrez-Rosado T, Rodríguez-Rey R, et al. Adaptation and psychometric properties of the Spanish Version of Child and Youth Resilience Measure (CYRM-32). Front Psychol 2019;10:1410; doi: 10.3389/fpsyg.2019.01410 31316419 PMC6610767

[52] Russell BS, Collins CM, Tomkunas AJ, et al. Exploring the factor structure of the Child and Youth Resilience Measure (CYRM-12) for Young children in a disadvantaged community. Child Youth Serv Rev 2021;120:105746; doi: 10.1016/j.childyouth.2020.105746

[53] Buckingham SL, Brodsky AE. Relative privilege, risk, and sense of community: Understanding Latinx immigrants' empowerment and resilience processes across the United States. Am J Community Psychol 2021;67(3–4):364–379; doi: 10.1002/ajcp.12486 33350477

[54] Bartoszek LA, Jacobs W, Unger JB. Correlates of resilience in Hispanic young adults. Fam Community Health 2020;43(3):229–237; doi: 10.1097/FCH.0000000000000261 32427670

[55] Rios M, Friedlander S, Cardona Y, et al. Associations of parental monitoring and violent peers with Latino youth violence. J Immigrant Minority Health 2020;22(2):240–248; doi: 10.1007/s10903-019-00894-6 31089909

[56] Shetgiri R, Kataoka SH, Ryan GW, et al. Risk and resilience in Latinos: A community-based participatory research study. Am J Prev Med 2009;37(6 Suppl 1):S217–S224; doi: 10.1016/j.amepre.2009.08.001 19896022

[57] Germán M, Gonzales NA, Dumka L. Familism values as a protective factor for Mexican-origin adolescents exposed to deviant peers. J Early Adolesc 2009;29(1):16–42; doi: 10.1177/0272431608324475 21776180 PMC3138713

[58] Roncancio AM, Ward KK, Berenson AB. Hispanic women's health care provider control expectations: The influence of fatalism and acculturation. J Health Care Poor Underserved 2011;22(2):482–490; doi: 10.1353/hpu.2011.0038 21551928 PMC3260793

[59] Yamanis T (Nina) J, Morrissey T, Bochey L, et al. “Hay Que Seguir En La Lucha”: An FQHC's community health action approach to promoting Latinx immigrants' individual and community resilience. Behav Med 2020;46(3–4):303–316; doi: 10.1080/08964289.2020.1738320 32701390

[60] U.S. Census Bureau. We the American…Hispanics. We the American. U.S. Census Bureau: Washington D.C.; 1993.

[61] Saenz R. Latinos and the Changing Face of America. Population Reference Bureau: Washington D.C.; 2004.

[62] McKnight-Eily LR, Okoro CA, Strine TW, et al. Racial and ethnic disparities in the prevalence of stress and worry, mental health conditions, and increased substance use among adults during the COVID-19 pandemic—United States, April and May 2020. MMWR Morb Mortal Wkly Rep 2021;70(5):162–166; doi: 10.15585/mmwr.mm7005a3 33539336 PMC7861483

[63] Andrade N, Ford AD, Alvarez C. Discrimination and Latino health: A systematic review of risk and resilience. Hisp Health Care Int 2021;19(1):5–16; doi: 10.1177/1540415320921489 32380912

[64] Cobb CL, Salas-Wright CP, John R, et al. Discrimination trends and mental health among native- and foreign-born Latinos: Results from national surveys in 2004 and 2013. Prev Sci 2021;22(3):397–407; doi: 10.1007/s11121-020-01186-4 33231824 PMC10371212

[65] Rabin J, Stough C, Dutt A, et al. Anti-immigration policies of the Trump administration: A review of Latinx mental health and resilience in the face of structural violence. Anal Soc Iss Public Policy 2022;asap.12328. [Epub ahead of print]; doi: 10.1111/asap.12328

